# Maximum carotid intima-media thickness and NT-pro BNP in association with retinal vein occlusion

**DOI:** 10.1371/journal.pone.0291456

**Published:** 2023-12-14

**Authors:** Hajime Onoe, Koji Tanaka, Norihiro Tsuchiya, Keisuke Miyata, Mai Kitaoka, Mao Nakayama, Ryusaburo Mori, Hiroyuki Nakashizuka

**Affiliations:** 1 Division of Ophthalmology, Nihon University School of Medicine, Tokyo, Japan; 2 Omotesando Internal Medicine and Ophthalmology Clinic, Tokyo, Japan; Osaka University of Pharmaceutical Sciences, JAPAN

## Abstract

The purpose is to clarify the relationship between patients with retinal vein occlusion (RVO), maximal intima-media complex thickness (Max IMT), and N-terminal pro-brain natriuretic peptide (NT-proBNP), which is useful in assessing atherosclerosis. This was a retrospective observation, single center study. The patients were 86 RVO patients (male: female = 43:43, mean age 63.3 years), 25 with central retinal vein occlusion (CRVO) and 61 with branch retinal vein occlusion (BRVO), classified as ≧50 years old and <50 years old, Max IMT≧1.1 and less, NT-pro BNP≧55 and less. Results showed that Max IMT ≧1.1 was significantly more common in both the CRVO and BRVO groups at ≧50 years, and NT-pro BNP ≧55 was significantly more common in the CRVO group. Max IMT≧1.1 was seen in 80% of the BRVO group and in 85% of patients aged ≧50 years. Sixty-eight percent of patients in the CRVO group had Max IMT≧1.1, but none of those < 50 years had Max IMT≧1.1. Forty-eight percent of RVO patients had NT-pro BNP≧55, and significantly more patients had Max IMT≧1.1 than those who did not have NT-pro BNP more than 55 (p = 0.02). Multiple regression analysis with Max IMT as the dependent variable showed that age and NT-pro BNP were significantly associated with RVO (p = 0.015, 0.022). RVO patients were more likely to have a Max IMT≧1.1, which was associated with atherosclerosis. Max IMT and NT-pro BNP were also associated with RVO patients, so NT-pro BNP may be a marker of RVO.

## Introduction

Retinal vein occlusion (RVO) has been reported to be associated with atherosclerotic cerebral and cardiovascular diseases, as well as with their risk factors such as hypertension, dyslipidemia, diabetes mellitus and smoking [1–4]. In the development of branch retinal vein occlusion (BRVO), venous thrombi occur at the retinal arteriovenous crossing. The arteriovenous crossing phenomenon is also a criterion for determining arteriosclerosis in the Scheie classification, suggesting RVO is related to arteriosclerosis [5]. Recently, patients with RVO were reported to have significantly higher LDL cholesterol and brachial-ankle pulse wave velocity (baPWV), and significantly more carotid plaques [6]. However, reports examining the relationship between RVO, and the presence of atherosclerosis are rare.

One indicator of arteriosclerosis is the maximal intima-media complex thickness (max carotid intima-media thickness; Max IMT) on carotid ultrasonography [7–9]. In Japan, the standard value of Max IMT for the presence of atherosclerosis is defined as 1.1 mm or more [10, 11]. Although Max IMT has been associated with coronary artery disease and thromboembolic stroke [12], few reports have examined the association between RVO and Max IMT [13].

While the prevalence of hypertension is high in BRVO [1], which is strongly associated with atherosclerosis, hypertension has not been associated with central retinal vein occlusion (CRVO) in younger patients [14]. However, masked hypertension with normal office blood pressure and high home blood pressure exists [15], making it difficult to determine the presence of hypertension on an outpatient basis. CRVO has also been reported to be caused by inflammation that is not due to arteriosclerosis [16]. In RVO cases, it is also necessary to search for risk factors such as hypertension and other lifestyle diseases, and an underlying metabolic syndrome.

The diagnosis of hypertension requires consideration of masked hypertension, which occurs in 10–15% of patients with normal blood pressure in the examination room, and blood pressure control status. Recently, blood BNP, which is used to determine heart failure and its risk, has been used as a biomarker to search for risk factors for life-style diseases. The N-terminal fragment of human brain natriuretic peptide is a peptide that is released into the blood during the generation of BNP from the pre-cursor of brain natriuretic peptide (BNP). NT-pro BNP (N-terminal pro-brain natriuretic peptide: NT-pro BNP) is used as a biomarker for heart failure [17], and has diuretic effects, a decrease in vascular resistance, suppression of renin and aldosterone secretion, and inhibition of myocardial hypertrophy. NT-pro BNP is useful as a marker of lifestyle diseases, as a level of 55 mg/dL or higher is considered suspicious for lifestyle diseases such as hypertension [18]. In this study, Max IMT, which is an established marker of arteriosclerosis in internal medicine, and NT-pro BNP, a marker of heart failure, were examined in more detail in RVO cases and risk factors related to RVO.

## Materials and methods

This study adhered to the tenets of the Declaration of Helsinki. This was a retrospective, single center study, and the procedures were approved by the Ethics Committee of the Nihon University Hospital, Tokyo, Japan (Approval No. 20210801). Informed consent was obtained from all individual patients verbally and documented in the medical record. We had access to information that could identify individual participants during or after data collection. RVO patients who visited Nihon University Hospital from December 2019 to March 2021 and had IMT measured by carotid echocardiography and NT-pro BNP measured by blood sampling were included. A Max IMT >1.1 mm was defined as having atherosclerosis [8]. Hypertension, a risk factor for arteriosclerosis, was defined as receiving treatment or having an office blood pressure of 140/90 mmHg or higher, dyslipidemia as receiving treatment or having an LDL cholesterol of 140 mg/dL or higher, diabetes as receiving treatment or having an HbA1c of 6.5% and blood sugar of 200 mg/dL or higher at any time and smoking as having an active smoking habit. To separate RVO patients not associated with atherosclerosis from those associated with atherosclerosis, BRVO and CRVO patients were classified into two groups for Max IMT: those over and those under the age of 50 years, respectively. The two groups were examined for an association with risk factors for atherosclerosis such as hypertension, dyslipidemia, diabetes mellitus and smoking history. As NT-pro BNP is affected by age [19], we examined only the association with Max IMT.

### Equipment and methods for measuring Max IMT

Max IMT is defined as the maximum intima-media thickness of the proximal, distal and bilateral walls of the common carotid artery (CCA), carotid bulb (CB) and in-ternal carotid artery (ICA) in the area where it can be observed. Max IMT was measured using one of the following ultrasonographic instruments: LOGIQ E9 (GE Healthcare, Japan), LOGIQ E10 (GE Healthcare Japan), Aplio i800 TUS-AI800 (Canon Medical Systems Inc., Japan) or ALOKA ARIETTA 850 (Fujifilm Healthcare Corporation, Japan).

Initially, the conventional B-mode imaging of the extracranial CCA, the CB and the ICA in the neck was performed bilaterally and the carotid IMT was measured, as reported in previous studies [20]. The site of the greatest IMT including the plaque lesion was sought along the arterial wall and measured for each projection. We defined the greatest value among all projections as Max IMT.

### Statistical analysis

Results are reported as standard deviation (SD) for each group. The distribution of Max IMT and the association between Max IMT and NT-pro BNP in RVO patients were subjected to Fisher’s exact test. Univariate and multivariate linear regression analyses were performed to determine the factors associated with Max IMT. Max IMT was log-transformed to approximate a normal distribution. A P-value of <0.05 was considered a statistically significant difference, and all P-values were two-sided. All statistical analyses were performed using SPSS version 23.0 (IBM Japan, Ltd., Tokyo, Japan).

## Results

Characteristics of RVO patients are shown in Table 1. We included 61 eyes of 61 patients with BRVO, of whom 28 were male and 33 female, with a mean age of 64 ± 12.1 years. 53 patients were 50 years and older, whereas 8 were under the age of 50 years. We also included 25 eyes of 25 patients with CRVO, of whom 15 were male and 10 female, with a mean age of 62 ± 15.9 years. Nineteen patients were over 50 years old and 6 were under 50 years old. Hypertension was present in 80% of BRVO and 68% of CRVO, dyslipidemia in 50% of BRVO and 32% of CRVO, diabetes in 16% of BRVO and 24% of CRVO, smoking in 12% of BRVO and 36% of CRVO.

**Table 1:** Patient characteristics.

	BRVO	CRVO
Number (eyes)	61	25
Age (years)^a^	64±12.1	62±15.9
Males: females	28:33	15:10
Hypertension (+)	80%	68%
Dyslipidemia (+)	50%	32%
Diabetes mellitus (+)	16%	24%
Smoking history (+)	12%	36%

BRVO: Branch retinal vein occlusion

CRVO: Central retinal vein occlusion

^a^ Values are presented as the mean±standard deviation

Max IMT averaged 1.8±0.8 mm, 49 patients (80%) had Max IMT ≧1.1 mm, and 12 patients had Max IMT <1.1 mm in BRVO group. The mean Max IMT was 1.9±0.8mm, ≧1.1mm in 45 cases (85%), and <1.1mm in 8 cases over 50 years old.

Max IMT averaged 1.9 ± 1.1 mm, with 17 cases (68%) ≧ 1.1 mm and 8 cases < 1.1 mm in CRVO group. Mean Max IMT was 2.2 ± 1.0 mm, 17 patients (89%) over 50 years had an IMT greater than 1.1 mm, and 2 patients had an IMT of less than 1.1 mm. No patient under 50 years had an IMT greater than 1.1 mm (Table 2).

**Table 2:** Distribution of Max IMT in BRVO and CRVO patients.

		Max IMT (mm)^a^	Max IMT < 1.1mm (eyes)	Max IMT≧1.1mm (eyes)	≧1.1mm (%)	p-value
BRVO	< 50 years	1.4±0.7	4	4	50	p = 0.041
	≥ 50 years	1.9±0.8	8	45	85	
	Total	1.8±0.8	12	49	80	
CRVO	< 50 years	0.8±0.1	6	0	0	p<0.0001
	≧50 years	2.2±1.0	2	17	90	
	Total	1.9±1.1	8	17	65	

Max IMT: Max carotid intima-media thickness

BRVO: Branch retinal vein occlusion

CRVO: Central retinal vein occlusion

^a^ Values are presented as the mean±standard deviation

In the BRVO group, 29 patients (47%) had NT-pro BNP ≥55 pg./mL, of whom 25 had Max IMT ≥1.1 mm (25/29 = 86%). In the CRVO group, 12 patients (48%) had NT-pro BNP ≥ 55 pg/mL. Of these, 11 (11/12 = 92%) had a Max IMT≧1.1 mm. Total retinal vein occlusion patients and CRVO group, Max IMT > 1.1 and NT-pro BNP > 55 were significantly associated (p = 0.02), but not for BRVO group (Table 3).

**Table 3:** Relationship between Max IMT and NT-pro BNP in RVO patients.

		NT-pro BNP≧55	NT-pro BNP<55	p-value
BRVO	Max IMT≧1.1	25	24	p = 0.34
	Max IMT <1.1	4	8	
CRVO	Max IMT≧1.1	11	6	p = 0.02
	Max IMT<1.1	1	7	
Total RVO	Max IMT≧1.1	36	30	p = 0.02
	Max IMT<1.1	5	15	

NT-pro BNP: N-terminal pro-brain natriuretic peptide

Max IMT: max carotid intima-media thickness

BRVO: Branch retinal vein occlusion

CRVO: Central retinal vein occlusion

Multiple regression analysis with Max IMT as the dependent variable showed an association in the order of age and NT-pro BNP for RVO overall (p = 0.015, p = 0.022) (Table 4).

**Table 4:** Multiple regression analysis with Max IMT as dependent variable of RVO.

	univariate analysis					
	Regression coefficient	SE	β	t-value	P-value	
age (per year)	0.020	0.003	0.542	5.918	**p<0.0001**	
male sex	0.052	0.105	0.053	0.490	0.625	
History of hypertension	0.152	0.124	0.133	1.227	0.223	
History of diabetes	0.211	0.133	0.170	1.579	0.118	
History of dyslipidemia	0.144	0.105	0.148	1.375	0.173	
History of smoking	0.153	0.111	0.149	1.380	0.171	
ln_NT-pro BNP	0.212	0.044	0.465	4.811	**P<0.0001**	
	multivariate analysis					
	Regression coefficient	SE	β	t-value	P-value	VIF
age (per year)	0.011	0.005	0.313	2.476	**0.015**	2.008
male sex	-					
History of hypertension	-					
History of diabetes	0.223	0.116	0.180	1.926	0.058	1.098
History of dyslipidemia	0.073	0.089	0.075	0.824	0.412	1.050
History of smoking	0.109	0.095	0.106	1.154	0.252	1.063
ln_NT-pro BNP	0.133	0.057	0.293	2.331	**0.022**	1.982

SE: standard error

VIF: variance inflation factor

NT-pro BNP: N-terminal pro-brain natriuretic peptide

Max IMT: max carotid intima-media thickness

BRVO: Branch retinal vein occlusion

CRVO: Central retinal vein occlusion

**Fig 1:**
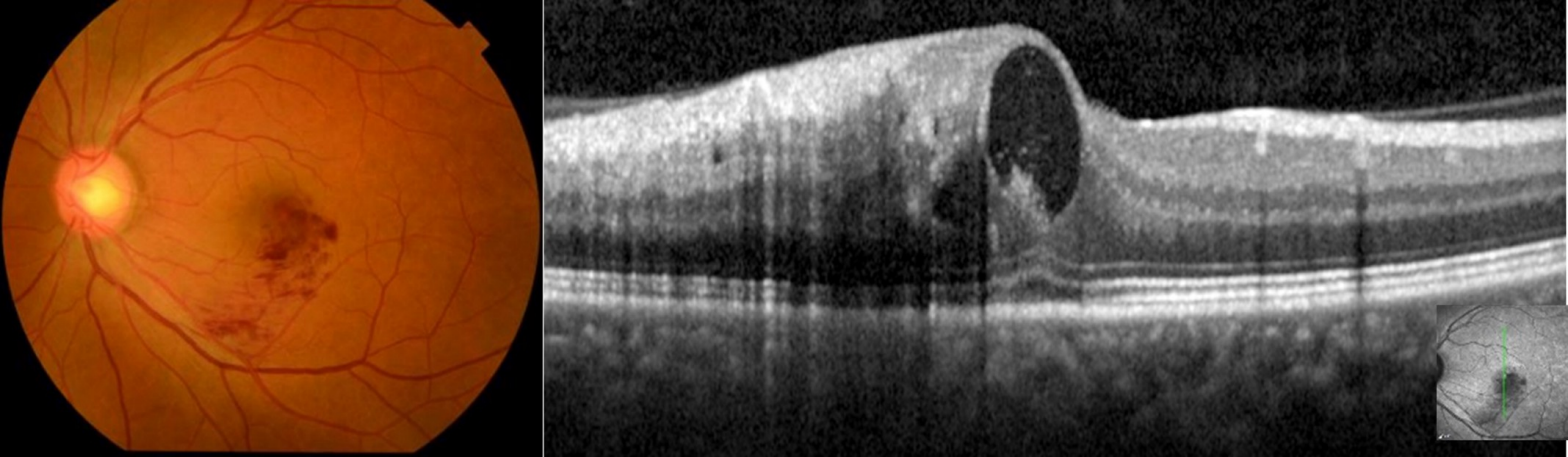
Representative case: 50-year-old male (1). (Upper left) A color fundus photograph showing retinal hemorrhage. (Upper right) OCT image shows macular edema.

**Fig 2:**
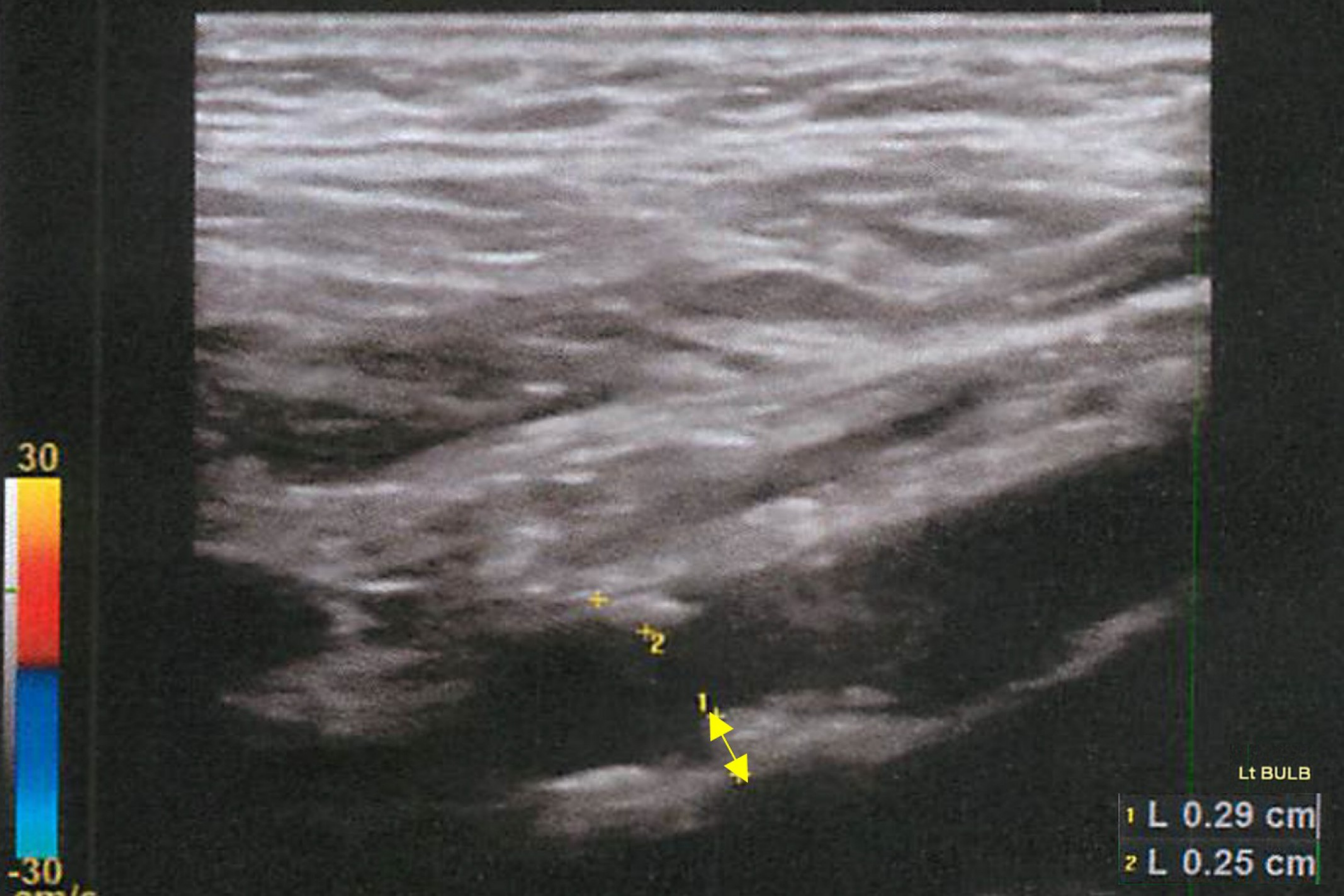
Representative case: 50-year-old male (2). IMT image of a carotid artery echo. Yellow arrows indicate plaques. In this patient’s case, the plaque in the left carotid sinus was the thickest, with a Max IMT of 2.9 mm.

Representative cases are shown in Figs 1 and 2. The case is 50-year-old male, BRVO and NT-proBNP is 43.

## Discussion

Carotid ultrasound measurement of Max IMT is noninvasive and reproducible and is recommended in guidelines as a standard for evaluating atherosclerosis [7]. Although an increased Max IMT has been associated with coronary artery disease and thromboembolic stroke [8], no association has been reported in retinal vein occlusion, which is thought to be related to atherosclerosis. The mean age of RVO patients in this study was 63 years, and 77% of cases had a Max IMT≧1.1 mm. In comparison, in a cohort of normal subjects, thickened IMT was reported in 20% of 63-year-olds [21], suggesting that RVO patients have a high rate of thickened Max IMT.

BRVO was originally thought to be caused by a venous compression by a thickened arteriole at an arteriovenous crossing site due to atherosclerosis, which would support the fact that Max IMT is higher in RVO patients. Hypertension, smoking, serum cholesterol, diabetes mellitus and aging have been reported as risk factors for arteriosclerosis [22], but there are few serum markers for arteriosclerosis itself. BNP was previously used as a marker of myocardial overload and heart failure. In recent years, however, NT-pro BNP has been used as an indicator of cardiac function due to its lack of bioactivity and longer half-life compared to BNP, which is why it is detectable in both plasma and serum and is not affected by hemolysis [17]. NT-pro BNP is not only an indicator of cardiac function but is also related to systolic blood pressure and LDL cholesterol. BNP seems to be significantly higher in patients with abnormal systolic blood pressure compared to healthy subjects [23]. Thus, NT-pro BNP is thought to be related to hypertension.

In the present study, an association between Max IMT and NT-pro BNP was found in RVO patients and in CRVO patients. This may be because Max IMT is a well-established indicator of arteriosclerosis, resulting in an association between Max IMT and NT-pro BNP. In contrast, no association was found between Max IMT and high NT-pro BNP in BRVO. Among the 24 patients with Max IMT > 1.1 and NT-proBNP < 55, 8 were < 50 years old. Because NT-proBNP is age-dependent, the BRVO may showed no association. However, when multiple regression analysis was performed that added other factors such as age, NT-pro BNP was associated with RVO. This may be due in part to the fact that Max IMT increases with age [24]. In addition, none of the patients in the CRVO group below 50 years had a Max IMT ≥1.1, while 90% of patients in the CRVO group above50 years had a Max IMT ≥1.1. These results suggest that the pathophysiology of CRVO may differ after the age of 50 years. Previous reports have indicated that psychological and physical stress can contribute to the onset of CRVO in patients under 50 years old [25]. BNP is known to be secreted under sympathetic nervous system stimulation, which opens up the possibility of a correlation with NT-pro BNP in CRVO patients under 50 years old.　In contrast, 50% of patients with BRVO had Max IMT ≥1.1, even if they were younger than 50 years. Given that Max IMT is age-dependent, BRVO may be strongly associated with atherosclerosis.

This is the first report to show that Max IMT and NT-pro BNP are associated with RVO patients. In conclusion, a Max IMT ≥1.1 and NT-pro BNP ≥55 was associated with RVO patients and as such, they may represent useful biomarkers. Elevated values of these markers may be a good indication for consulting a physician, since they have been as-sociated with systemic diseases such as coronary artery disease and thromboembolic stroke. Max IMT may increase but not decrease, but carotid artery echocardiography cannot be performed in the ophthalmologist’s office and should be requested. NT-pro BNP, on the other hand, can be used in RVO patients as a simple marker in blood. However, NT-pro BNP levels can vary and should be interpreted with caution.

Limitations of this study include the small number of participants and the lack of controls. Further studies are expected in the future.

## Supporting information

S1 Data All data included in this file. (XLSX)
